# PRESAGE: PRivacy-preserving gEnetic testing via SoftwAre Guard Extension

**DOI:** 10.1186/s12920-017-0281-2

**Published:** 2017-07-26

**Authors:** Feng Chen, Chenghong Wang, Wenrui Dai, Xiaoqian Jiang, Noman Mohammed, Md Momin Al Aziz, Md Nazmus Sadat, Cenk Sahinalp, Kristin Lauter, Shuang Wang

**Affiliations:** 1 Department of Biomedical Informatics, University of California San Diego, La Jolla, 92093 CA USA; 2 Department of Computer Science, Syracuse University, Syracuse, 13244 NY USA; 3 Department of Computer Science, University of Manitoba, Winnipeg, R3T 2N2 MB Canada; 4 Department of Computer Science and Informatics, Indiana University, Bloomington, 47408 IN USA; 5 Cryptography Group, Microsoft Research, San Diego,, 92122 CA USA

**Keywords:** SGX, Homomorphic encryption, Data outsourcing, Privacy preserving

## Abstract

**Background:**

Advances in DNA sequencing technologies have prompted a wide range of genomic applications to improve healthcare and facilitate biomedical research. However, privacy and security concerns have emerged as a challenge for utilizing cloud computing to handle sensitive genomic data.

**Methods:**

We present one of the first implementations of Software Guard Extension (SGX) based securely outsourced genetic testing framework, which leverages multiple cryptographic protocols and minimal perfect hash scheme to enable efficient and secure data storage and computation outsourcing.

**Results:**

We compared the performance of the proposed PRESAGE framework with the state-of-the-art homomorphic encryption scheme, as well as the plaintext implementation. The experimental results demonstrated significant performance over the homomorphic encryption methods and a small computational overhead in comparison to plaintext implementation.

**Conclusions:**

The proposed PRESAGE provides an alternative solution for secure and efficient genomic data outsourcing in an untrusted cloud by using a hybrid framework that combines secure hardware and multiple crypto protocols.

## Background

The advance of sequencing technology has significantly lower the costs of generating genomic data for improving healthcare, discovering new treatment methods and facilitating biomedical research [1]. For example, Precision Medicine Initiative (PMI) [2] aims to usher in a new era of medicine by collecting genomic data from a million people, by which more targeted treatment could be developed. It is becoming a big challenge to efficiently store and process the huge amount of genomic data in biomedical research [3]. Recently, cloud computing emerges [4] as an ideal platform for providing elastic computation and storage resources for genomic data analysis. However, privacy concerns [5, 6] have posed challenges to outsource genomic data in an untrusted cloud environment. Individual genomic information tends to reveal sensitive personal information including, but not limited to, personal identity [7, 8], disease condition [9–11], appearance [12]. As genomic data are shared by blood relatives, the dissemination of personal genomic information may have negative impact on other family members [13, 14]. For example, Lin et al. [15] demonstrated that a number of 75 statistically independent SNPs may be enough to re-identify an individual. Sweeney et al. [8] demonstrated that 84-97% patients profiles in the Personal Genome Project (PGP) could be identified by linking their demographic information to publicly available records. Gymrek et al. [7] illustrated that surname inferences for U.S. males could be performed by matching Y-chromosome haplotypes in recreational genetic genealogy databases. Claes et al. [12] modeled the 3D human facial appearance based on gender, genomic ancestry, genotype and specific genes that determine facial features. Furthermore, sensitive patient information would also be recovered from aggregated statistics [9, 16, 17]. By utilizing the reference population from the International HapMap Project, Homer’s attack model [9] is able to re-identify individuals in a case group from the aggregated allele frequencies in genome-wide association studies (GWAS). A recent study by Shringarpure et al. [16] demonstrated that even binary query results (i.e., existence of variants) from the genomic data sharing Beacon project [18] can still reveal sensitive personal information.

To protect the privacy and confidentiality of genomic data, many cryptographic methods have been developed. Homomorphic encryption (HME) is one of the most popular technologies for secure computation over the encrypted data. Since the first fully HME scheme was proposed by Gentry [19] to support both addition and multiplication operations over encrypted data, the performance of HME technology has been improved significantly [20–22]. Many HME-based applications have been studied for safeguarding linear classification [23], predictive analysis on encrypted medical data [24], genetic association studies [25, 26], Edit distance computation [27], GWAS study using exact logistic regression [28]. Secure multiparty computation (SMC) is another widely adopted technique for securing genomic data analysis, such as secure multiparty GWAS [29–33], secure distributed regression model learning [34] and so on. However, the high computational complexity of the existing HME and SMC solutions plague their practical adoption over the large-scale genomic data.

Recently, Software Guard Extension (SGX) [35] has been released to be an alternative solution for securing computation over sensitive data by using a hybrid system combining both secure hardware and software. It allows an application to create a protected container, namely enclave, to guarantee integrity and confidentiality of sensitive data and computation under the protection against potential privileged softwares. A detailed discussion of SGX can be found in the overview of SGX section. Many studies have demonstrated the feasibility of applying SGX as efficient solutions for secure and privacy-preserving computation in cloud computing [36, 37], ancestry analysis [38], international collaboration on rare disease analysis [39]. Thus, in this paper, we proposed an SGX based framework to enable both secure and efficient outsourcing of genetic testing in an untrusted cloud environment.

Genetic testing has become affordable and ubiquitous with the development of whole genome sequencing technology. It would potentially benefit healthcare by providing clinical decision support and prognostic estimates for patients and their related subpopulation, e.g., supporting diagnosis of disease, determining personalized medicine and treatment and evaluating the risk of disease. Genetic testing matches the targeted biomarkers to identify the variations in chromosome, gene and proteins. Although data owners can efficiently perform genetic testing by outsourcing the storage and computation to cloud services, the liability of genomic data security and privacy is still a major concern. Many efforts have been attempted to provide better protection for genetic test. For example, in [40], a privacy-preserving toolkit named GenoDroid is proposed for genomic tests like paternity testing, ancestry testing and personalized medicine testing. Another secure primitive [41] was developed based on additively homomorphic encryption to outsource genetic testing without revealing the sizes and positions of biomarkers to be matched. Danezis et al. [42, 43] proposed two cryptographic protocols to evaluate private disease susceptibility with a weighted combination of the targeted genetic markers. Another privacy-preserving genetic testing framework [44] is proposed based on homomorphic encryption to make HIV-related prediction. De Cristofaro et al. [45] developed yet another a privacy-preserving protocol to allow a cloud server to securely perform genetic relatedness test on encrypted genomic data. As mentioned above, most of existing secure genetic testing frameworks are facing the scalability issues due to the high computation overhead. In this paper, we explore an alternative SGX based solution to enable both secure and efficient outsourcing genomic data storage and computation on an untrusted cloud for the purpose of genetic testing. The main contributions of the proposed studies are as follows:

- We present one of the first implementations of SGX based secure genetic testing framework to facilitate efficiently outsourced storage and computation. The secure outsource storage is achieved through data sealing scheme within SGX framework, which is immune to replay attack.
- We have taken into account the oblivious access protection by using 4KB page-wise data access model.
- To improve the performance, we adopt a perfect hashing scheme to achieve *O*(1) complexity data access within each 4KB page.

### Overview of software guard extension

Software Guard Extensions (SGX) [46] is a security extension of Intel processor architecture. SGX tends to provide security and confidentiality guarantee for secure computing on hosts. By using SGX, privileged modules like operating system (OS), virtual machine (VM) scheduler etc. are isolated from private codes and secret data through hardware protection. More specifically, instead of quarantining malicious parts within the running system as traditional security sandbox, Intel SGX uses the “inverse sandbox” design to seal private codes, sensitive data and other selected secrets into a CPU secure computation unit called “Enclave”. The access of secrets within enclaves are strongly restricted by the hardware supported access control. This fact implies that Intel SGX can effectively protect secrets for applications, even though the other privileged parts are attacked and compromised by malicious components.

**Fig. 1:**
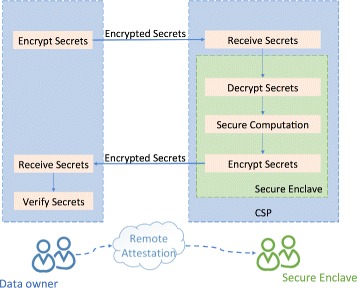
Overview of a typical SGX framework. It consists of data owner, untrusted cloud service provider, and secure enclave

An overview of a typical SGX framework is illustrated in Fig. 1. A typical SGX based application consists of data owner, untrusted cloud service provider (CSP), and the secure enclave. First, the data owner establishes a secure channel with the enclave hosted by an untrusted CSP through the remote attestation process [46]. Then, the data owner can securely upload data to the CSP (data provisioning). In SGX, all decrypted secrets can only be accessed by the authorized codes, which also lie inside the enclave. A hardware supported access control proxy guarantees the code and data cannot be accessed or modified by softwares outside the secure enclave. It is quickly becoming a hot area of study. Recent investigations have demonstrated the potential of SGX to improve the security and privacy in real-world applications including shielded execution of server applications [47] and trustworthy data analytics [36] in the cloud, secure execution environment for network applications [48], secure function evaluation [49], and oblivious multi-party machine learning [50]. In these applications, SGX provided hardware-level security guarantees with a reduced computational complexity comparing with the traditional cryptographic methods. In this paper, we propose a SGX-based method to enable securely outsourced genetic testing.

## Methods

**Fig. 2:**
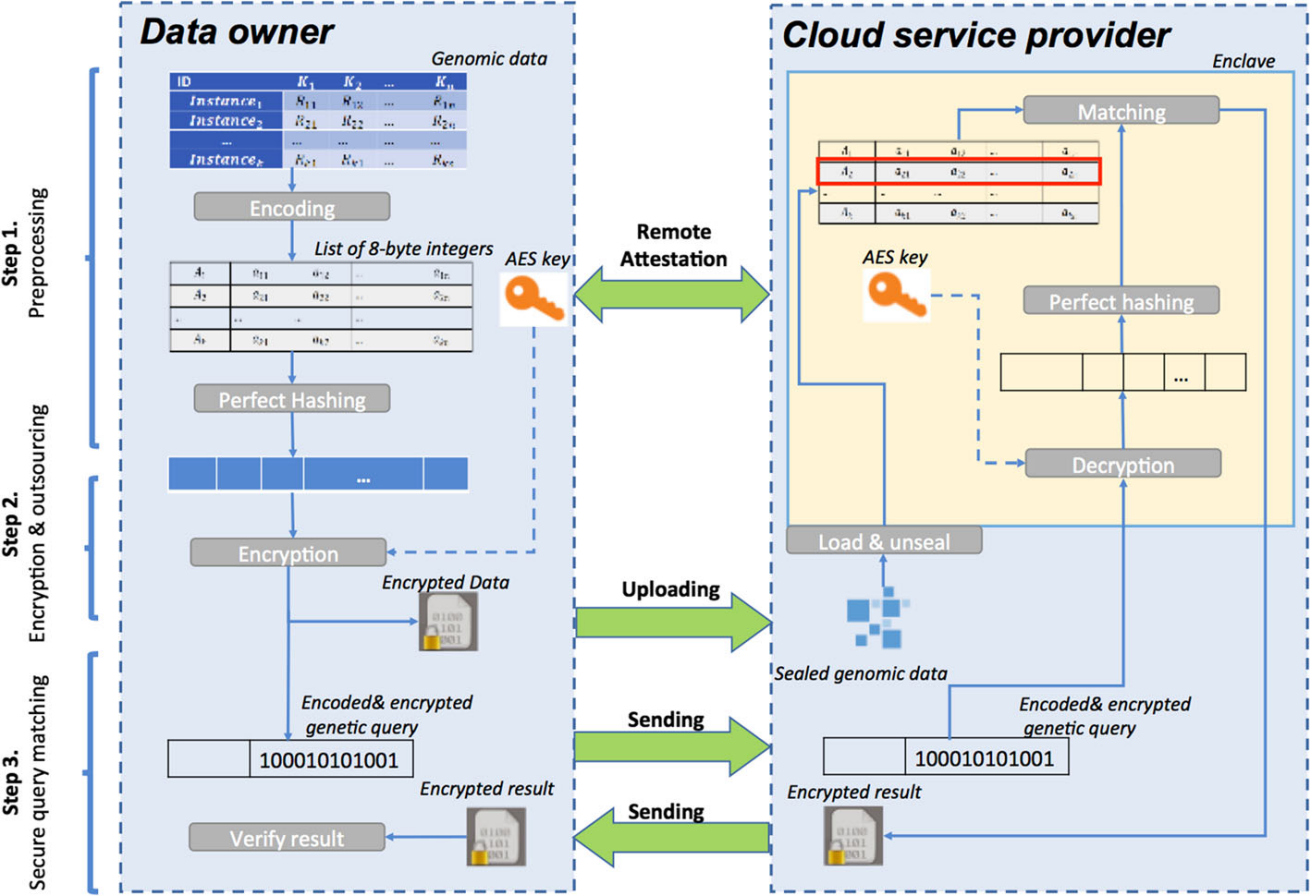
Workflows of the proposed PRESAGE framework. It presents in three consecutive steps

**Fig. 3:**
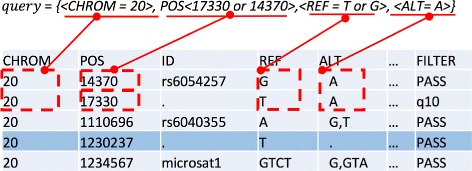
A sample query for retrieving count of records from VCF files

In this section, we present the proposed PRESAGE framework for securely outsourcing genetic tests using SGX. Figure 2 provides an overview of the proposed PRESAGE framework. Our framework is optimized for the dual objectives of security and efficiency. In our proposed framework, we support genomic queries, which count genomic records by matching a set of biomarkers in the VCF files. More specifically, the attributes to be matched include chromosome ID (CHROM), position (POS), reference (REF) and alternative alleles (ALT). Figure 3 shows a sample query, where a query consists of 4 tuples, and each tuple indicates certain matching conditions. In this example, the query will locate all records in a VCF file that meet the conditions of CHROM = 20, POS = 17330 or 14370, REF = ‘T’or ‘G’ and ALT = ‘A’. The results of this query will the count of matched records (i.e. 2 in this example). This query is equivalent to a Structured Query Language (SQL) query as follow:

***SELECT*** *count(∗)* ***FROM*** *sample.vcf* ***WHERE*** *CHROM = 2* ***AND*** *(POS = 17330 or 14370)* ***AND*** *(REF= ‘T’ or ‘G’)* ***AND*** *ALT = ‘A’.*

In the remaining part of this section, our approach will be introduced in details.

**Fig. 4:**
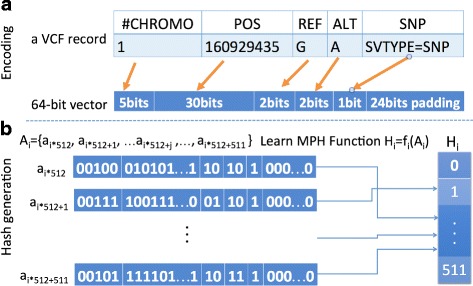
Workflow of the proposed encoding and MPH procedures. **a** Encoding, **b** Hash generation

**Step 1.** Preprocessing. We assume that a data owner holds a private genomic database in Variant Call Format (VCF). The goal of data preprocessing is to minimize the potential overheads for the outsourced matching process. Genomic records in the VCF file is sparse, which only contains a few million variants in comparison to the whole genome with 3 billion base pairs. In order to represent the spare VCF records more efficiently, we use minimal perfect hash (MPH) to map n input records into n consecutive integers, by which each input record can be accessed at a constant time. For example, data owner has a dataset *R*={*r*_1_,*r*_2_,…,*r*_*n*_}, where *r*_*j*_ denotes a single record, and n is the total number of records. For each record *r*_*i*_ in the VCF file, data owner encodes the fields of #CHROM with 5 bits, POS (i.e., reference position) with 30 bits, REF (i.e., reference alleles) with 2 bits, ALT (i.e., alternative alleles) with 2 bits and SNP flag with 1 bit into a 40-bit vector based on the characteristics of human genome [51]. To improve access efficiency, this 40-bit vector will be stored in the first 40-bit of a 64-bit/8-byte integer with the rest bits of the integer as 0s. By this data alignment (widely used in modern software design), each record can be retrieved within one instruction in the x86 architecture. Let us denote by ai the 64-bit integer. Then *A*={*a*_0_,*a*_1_,*a*_2_,…,*a*_*n*−1_} is a list of encoded integers. The data owner can learn a MPH function denoted by *h*_*j*_=*f*(*a*_*j*_), where the unique hash hi is an integer ranging from 0 to *n*−1. More specifically, the FCH algorithm proposed by Fox, Chen and Heath [52] was used in our PRESAGE framework. The FCH is very compact and efficient for small dataset (for PRESAGE, the whole dataset is divided into equal sized 500 records to protect paging pattern attack. See the last subsection of this part for more details). The generated hash from FCH algorithm can be stored in approximately 4.1 bits per key. Figure 4 shows the workflow and encoding and MPH generation.

**Step 2.** Encryption and data outsourcing. A remote attestation procedure is required between the data owner and the enclave so that they can provide the evidences to prove their integrities and authenticities through the Elliptic Curve Digital Signature Algorithm (ECDSA) [53] and a quoting enclave. Once the attestation step is passed, data owner negotiates a session key with the enclave via the Elliptic curve Diffie–Hellman (ECDH) [54] protocol. Given the MPH function learned in step 1, each record in the VCF file will be encoded and reordered based on the hashing index followed by a data encryption step using Advance Encryption Standard-Galois/Counter Mode (AES-GCM) [55] for the sake of efficiency, secrecy and integrity. Then, the encrypted data will be uploaded to the CSP. A time varying initial vector will be used for encrypting each data block in AES to avoid the replay attack [56]. In addition, message authentication code (MAC) will be sent along with each encrypted message to ensure that the message are from the stated sender (i.e., authenticity) and has not been changed during transit (i.e., integrity). After receiving the encrypted data and hashed table, enclave seals them outside for long term storage and answering further queries from data user. Since the data are stored outside the enclave, the untrusted CSP may maliciously reorder data or provide the old versions to enclave for unsealing, which can be considered as a replay attack. To mitigate this kind of attack, we will embed additional MAC, timestamp and data owner information along with the sealed data.

**Step 3**. Secure Genetic Query Matching. Firstly, the data user will attest the remote enclave to check the integrity of enclave, and build a secure channel with the enclave. Then, the data querying phase for identifying the existence of certain genetic variants, encrypted queries that encode the chromosome #, position, reference and alternative alleles, will be sent to the CSP. Once the query is received, the enclave will unseal the data and hash functions stored in Enclave for query execution: a potential position (hash value) is obtained by applying hash function to query value. Finally, the enclave will encrypt the number of matching queries as result and send it back to the authorized data user. The above procedures ensure the data security and integrity for outsourced cloud based genetic testing.

## Results

### Experimental setup

The sizes of VCF datasets used in our experiments vary from 10,000 to 200,000 records. The data owner and CSP can communicate over a Secure Sockets Layer (SSL) channel, which is built based on OPENSSL library [57]. All of the experiments except the iDASH competition results are conducted on a Windows 10 SGX-enabled machine with i7 6820HK CPU and 48 GB memory. Both data owner and CSP were simulated on the aforementioned SGX machine. The iDASH competition results were evaluated on the Linux server with an Xeon Processor E3-1275 v5 and 64 GB memory [58]. All evaluation results of our PRESAGE framework are averaged over five trials.

### Experimental resultsp

Table 1 shows the runtime results of the PRESAGE framework, which include the key steps such as remote attestation, SNPs coding, hash generation, enclave creation, data sealing and different number of queries. All of above steps except for querying step can be considered as one-time jobs. The attestation, SNPs coding and hash generation steps are profiled on the data owner side, while the rest steps are profiled on the CSP side. As we can see, for different input data sizes, the time consumption of attestation and enclave creation is stable. The time consumption of SNPs coding, hash generation, and data sealing increases linearly with the increase of input data size. Among all these key steps, MPH generation is the most time consuming step. In contrast, the querying step is highly efficient, which took the least time among all steps based on our testing datasets. We can see that there is a trade-off between adopting MPH for hash generation and query execution. It is worth emphasizing that the MPH generation will be only a one-time process, but the query execution would be highly frequent.

**Table 1:** The breakdown run time (in seconds) of the proposed PRESAGE framework

						Query #	
Size	Attestation	Coding	Generating hash	Creating enclave	Sealing	1	3
10 K	0.121	0.016	1.130	0.169	0.094	0.003	0.025
50 K	0.126	0.080	6.371	0.173	0.517	0.012	0.013
100 K	0.124	0.164	13.473	0.179	0.980	0.023	0.025
500 K	0.120	0.309	28.677	0.171	2.045	0.043	0.048

Table 2 depicts data size and the memory consumptions in MB for different VCF datasets. We can see that the amount of encoded data after hashing is about $\frac {1}{6}$ size of these original VCF files. The sealed data imposed about $\frac {1}{3}$ overhead in storage due to the inclusion of the additional security information such as MAC to protect replay attack. The enclave memory usage is stable in PRESAGE framework for different setups, as we divided the large inputs into 4 KB page-wise block to process, which allows oblivious memory access in an efficient manner.

**Table 2:** The data size and enclave memory consumption (in MB) for different datasets

				Query #	
Size	Plaintext	Encoded data	Sealed data	Single query	3 queries
10 K	0.55	0.09	0.12	3.006	3.016
50 K	2.59	0.45	0.59	3.010	3.010
100 K	5.26	0.90	1.15	3.010	3.010
500 K	10.5	1.75	2.31	3.010	3.010

**Fig. 5:**
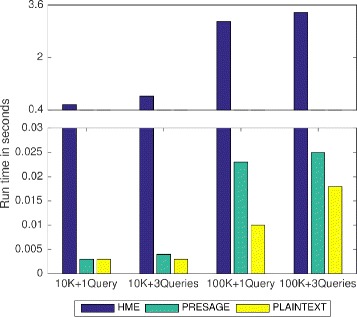
Comparison of querying performance among PRESAGE, HME-based method and plaintext implementation

Figure 5 is the comparison between the SGX and plaintext. Following the standards of the the iDASH 2016 genome privacy competition [58], we implemented the PRESAGE over 10K and 100K SNPs sizes. To benchmark the performance of the plaintext, we also implemented the same query algorithm outside the enclave. As we can see, the PRESAGE is about 120 times faster than the HME-based method [58] as reported in the 2016 genome privacy protection competition [59]. However, PRESAGE still showed some computational overhead in comparison to plain text based implementation due to the extra data unsealing steps and memory encryption in SGX.

## Discussion

### Security model

The proposed PRESAGE framework is designed under the assumption of a malicious CSP, which may deviate arbitrarily from their predefined protocols. The CSP has full control over the hardware and software environments, which include the control of OS, VM, and all code invoked outside the SGX enclave. The malicious activities aim to break the confidentiality and integrity of the proposed framework. Some existing threats such as crashing the CPU hardware and interrupting the enclave execution are not considered in this paper [35, 60]. We try to minimize the controlled-side channel attacks due to the observation of page faulty access pattern through page-wise data blocking. In addition, we assume that the data owner fully trusts the design and correct implementation of secure enclave on the CPU hardware and SGX instructions. In PRESAGE, although the secure enclave is hosted by an untrusted CSP, the remote attestation step ensures to identify a trustworthy enclave and build a secure channel between the data owner/users and the enclave. The adoption of a 128 bits AES-GCM encryption protocol ensures a high-level security and integrity guarantee of all encrypted and sealed data. For storage and computation efficiency concerns, each record is encoded into a 40-bit vector and stored as an 8 byte integer, by which the amount of data operated in communication and sealing phases can be reduced dramatically. The minimal perfect hash is utilized to enable O(1) complexity data query in each page block. To avoid the paging access attack, we equally divided input data into 500 records to fit a 4 KB page-wise block in SGX. To enable secure data storage outsourcing, the seal data have been added with MAC in order to defend the replay attack. The sealed data only introduced about 31% storage overhead on average in our experiments.

### Limitation

There are several limitations of the proposed PRESAGE framework. First, the available Enclave Page Cache (EPC) for a single SGX machine is limited to 128 MB. Although, the enclave memory could be extended to 4 GB with software paging technique under Linux OS, it will impose computational overhead and still cannot avoid expensive data sealing and unsealing processes when genomic datasets exceed 4 GB. Some previous studies [35, 61] have identified the potential vulnerabilities of straightforward SGX implementations due to the memory access patterns, cache timing, page faults, hyper-threading, etc. Although, the proposed framework can take into account the protection of memory access patterns by using page-wise oblivious data access algorithm, we have not tackled other potential vulnerabilities. Finally, the proposed framework is based on the FCH scheme to build the perfect hashing on a single thread, which imposed a significant overhead at the data owner side. More efficient hashing mechanism or multi-threading based parallel hash building schemes will be considered in our future work. Moreover, the current implementation of PRESAGE store each 40-bit vector using a 8-byte integer, which will result in 24-bit unused space for each record. Additional data compression step and better hashing scheme could be adopted to improve the encoding efficiency. The above limitations warrant the further investigation of SGX based secure genomic data analysis framework.

## Conclusion

This paper proposed a secure outsourcing framework, which can defend malicious attack. To improve the efficiency, an MPH scheme has been incorporated. To avoid paging based attack, the input data are divided into small pieces in order to be filled into one 4 KB page. The outsourced data are sealed by the enclave and stored in an untrusted cloud. Our experiment results demonstrated the efficiency of the proposed PRESAGE framework. For a VCF file with 200K records, the PRESAGE securely processes a query within 0.05 s, which includes file loading, unsealing and query matching. Compared with state-of-the-art HME solution, PRESAGE framework shows at least 120X performance gain.

## Abbreviations

AES: Advanced encryption standard
CSP: Cloud service provider
ECDH: Elliptic curve Diffie–Hellman
ECDSA: Elliptic curve digital signature algorithm
EPC: Enclave page cache
GCM: Galois/Counter Mode
GWAS: Genome-wide association study
HME: Homomorphic encryption
MB: Megabyte
MAC: Message authentication code
MPH: Minimal perfect hash
OS: Operating system
PGP: Personal genome project
PMI: Precision medicine initiative
SEAL: Simple encrypted arithmetic library
SGX: Software guard extensions
SMC: Secure multiparty computation
